# Mapping the T cell response to COVID-19

**DOI:** 10.1038/s41392-020-00228-1

**Published:** 2020-07-02

**Authors:** Junwei Li, Junhua Wang, Angray S. Kang, Pradeep Kumar Sacitharan

**Affiliations:** 1 College of Veterinary Medicine, Qingdao Agricultural University, Qingdao, 266109 China; 2 Centre for Oral Immunobiology and Regenerative Medicine, Institute of Dentistry, Barts and the London School of Medicine and Dentistry, Queen Mary University of London, London, E1 2AT UK; 3 The Institute of Ageing and Chronic Disease, University of Liverpool, Liverpool, L7 8TX UK; 4 Xi’an Jiaotong-Liverpool University, Department of Biological Sciences, #111 Ren’ai Road, Suzhou Industrial Park, Suzhou, Jiangsu Province 215123 P.R. China

**Keywords:** Infection, Infectious diseases

**A recent article by Grifoni et al. elegantly demonstrated the ability to measure and understand the human CD4**^**+**^**and CD8**^**+**^**T cell responses to SARS-CoV-2 infection.**^1^**These findings highlighted below gave new insights into the immunopathogenesis of COVID-19, the crossreactivity of the SARS-CoV-2 infections, the potential targets of T cells, and for vaccine design.**

COVID-19 is a deadly global pandemic that spread across the world in a short time frame.^2,3^ Currently it is unknown how T cells, which comprise a major part of the adaptive immune response, react to the virus.^4^ Understanding the T cell response to the SARS-CoV-2 (virus which causes COVID-19) can aid vaccine development and increase our understanding on immunopathogenesis of the disease. Furthermore, accessing T cells responses in affected and non-affected patients can inform us on protective immunity to the virus. The authors set out to address these shortcomings by examining the T cells responses in patients who had recovered from SARS-CoV-2 infection with COVID-19 disease and unexposed individuals.^1^

The research group previously developed the megapool (MP) approach to allow simultaneous testing of large numbers epitopes, in particular when sample size may be limiting.^5^ The group then analyzed the bloods from 20 adult patients who had recovered from COVID-19 disease and local healthy control donors. Using flow cytometry to broadly assess the immunological cellular profile of recovered COVID-19 patients the authors showed the frequency of CD3^+^ cells was slightly increased in recovered COVID-19 patients relative to non-exposed controls, while no significant differences overall were observed in the frequencies of CD4^+^ or CD8^+^ T cells between the two groups. This elegant approach showed recovered COVID-19 patients consistently generated a substantial CD4^+^ T cell response against SARS-CoV-2. The SARS-CoV-2−specific CD4^+^ T cells were functional, as the cells produced IL-2 in response to non-spike and spike MPs. Polarization of the cells appeared to be a classical TH1 type, as substantial IFNγ was produced while little or no IL-4, Il-5, IL-13, or IL-17α was expressed. Most importantly the studies showed on average ~50% of the detected response was directed against the spike protein, and ~50% was directed against the MP representing the remainder of the SARS-CoV-2 orfeome. This is of significance, since the SARS-CoV-2 spike protein is a key component of the vast majority of candidate COVID-19 vaccines currently under development. The authors also detected IFNγ^+^ SARS-CoV-2−specific CD8^+^ T cells in the majority of COVID-19 cases. The majority of IFNγ^+^ cells co-expressed granzyme B and a substantial fraction of the IFNγ^+^ cells expressed TNF, but not IL-10. These data demonstrated the majority of recovered COVID-19 patients generated a CD8^+^ T cell response against SARS-CoV-2.

The authors next asked the question of whether stronger SARS-CoV-2-specific CD4^+^ T cell responses were associated with higher antibody titers in COVID-19 cases. This is an important question because most protective antibody responses are dependent on CD4^+^ T cell help. The authors with keen insight examined spike-specific CD4^+^ T cells because the spike component of the virus is the primary target of SARS neutralizing antibodies. The data showed spike-specific CD4^+^ T cell responses correlated well with the magnitude of the anti-spike receptor binding domain (RBD) IgG titers. This part of the study also showed CD4^+^ and CD8^+^ T cell responses to SARS-CoV-2 were generally well correlated.

For the final set of experiments in the article, the authors synthesized sets of overlapping peptides spanning the entire open reading frame of SARS-CoV-2 and pooled them separately so that each pool would represent one polypeptide. By doing this the authors hoped to understand which antigens are targeted by CD4^+^ and CD8^+^ T cells, whether the corresponding antigens are the same or different, and how do they compare to the antigens currently considered for COVID-19 vaccine development. SARS-CoV-2-specific CD4^+^ T cell targets included SARS-CoV-2 ORFs spike, M, and N which accounted for accounted for 27, 21, and 11% of the total CD4^+^ T cell response, respectively. The data also indicated a somewhat different pattern of immunodominance for SARS-CoV-2 CD8^+^ T cell reactivity with spike protein accounting for ~26% of the reactivity, and N accounting for ~12%. These results concluded that CD8^+^ T cell crossreactivity exists but is less widespread than CD4^+^ T cell crossreactivity.

**Fig. 1:**
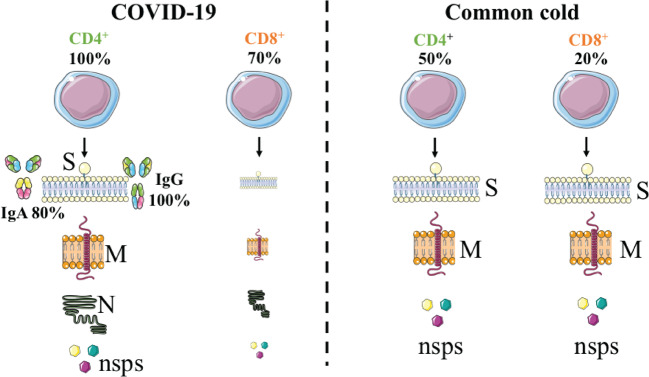
Measuring the T cell response to SARS-CoV-2 is critical for understanding COVID-19. Epitope pools detected CD4^+^ and CD8^+^ T cells in 100 and 70% of recovering COVID patients. T cell responses are focused not only on spike but also on M, N, and other ORFs. In addition, T cell reactivity to SARS-CoV-2 epitopes are also present in non-exposed individuals

In summary, Grifoni et al. provided critical knowledge that showed circulating SARS-CoV-2-specific CD8^+^ and CD4^+^ T cells were identified in ~70 and 100% of COVID-19 patients, respectively. CD4^+^ T cell responses to spike, the main target of most vaccine efforts, were robust and correlated with the magnitude of the anti-SARS-CoV-2 IgG and IgA titers. The authors also showed total CD4^+^ response was directed at the M, spike, and N proteins each accounted for 11–27% with additional responses commonly targeting nsp3, nsp4, ORF3a, and ORF8, among others. For CD8^+^ T cells, spike and M were recognized, with at least eight SARS-CoV-2 ORFs targeted. Importantly, the authors detected SARS-CoV-2−reactive CD4^+^ T cells in ~40–60% of unexposed individuals, suggesting cross-reactive T cell recognition between circulating ‘common cold’ coronaviruses and SARS-CoV-2. However, further studies are required on samples from acute patients and patients with complicated disease courses which will tell us the T cell responses for the whole duration of the disease. In addition, the common cold history and matched blood samples were not fully detailed for each patient. These data could provide us with more conclusions regarding the abundance of crossreactive coronavirus T cells before exposure to SARS-CoV-2. Moreover, further studies on the longevity of the SARS-CoV-2 immunological memory have to be conducted on samples from recovered patients. Nonetheless, Grifoni et al. provided the first key insights on how T cells respond to this deadly virus (Fig. 1).
